# Intussusception in Adults: The Role of MDCT in the Identification of the Site and Cause of Obstruction

**DOI:** 10.1155/2016/5623718

**Published:** 2015-12-27

**Authors:** Viola Valentini, Grazia Loretta Buquicchio, Michele Galluzzo, Stefania Ianniello, Graziella Di Grezia, Rosa Ambrosio, Margherita Trinci, Vittorio Miele

**Affiliations:** ^1^Department of Emergency Radiology, S. Camillo Hospital, Circonvallazione Gianicolense 87, 00152 Rome, Italy; ^2^Department of Radiology, Second University of Naples, Piazza Miraglia 2, 80138 Naples, Italy

## Abstract

Unlike pediatric intussusception, intestinal intussusception is infrequent in adults and it is often secondary to a pathological condition. The growing use of Multi-Detector Computed Tomography (MDCT) in abdominal imaging has increased the number of radiological diagnoses of intussusception, even in transient and nonobstructing cases. MDCT is well suited to delineate the presence of the disease and provides valuable information about several features, such as the site of intussusception, the intestinal segments involved, and the extent of the intussuscepted bowel. Moreover, MDCT can demonstrate the complications of intussusceptions, represented by bowel wall ischemia and perforation, which are mandatory to promptly refer for surgery. However, not all intussusceptions need an operative treatment. In this paper, we review the current role of MDCT in the diagnosis and management of intussusception in adults, focusing on features, as the presence of a leading point, that may guide an accurate selection of patients for surgery.

## 1. Introduction

Intestinal intussusception in adults is considered uncommon, accounting for an estimated 5% of all intussusceptions and representing only 1% of intestinal obstructions [1, 2]. Unlike pediatric intussusception, which is usually idiopathic, adult intussusception is most often secondary to an identifiable cause [3]. Many pathological conditions [4, 5], such as malignant or benign neoplasms, polyps, Meckel's diverticulum, and postoperative adhesions, may act as leading points by altering bowel peristalsis. Association with malignant tumors is more common in large bowel intussusception (65–70% of cases), while small bowel intussusceptions are secondary to a malignancy in 30–35% of cases only.

Before the widespread use of Multi-Detector Computed Tomography (MDCT), the diagnosis was based on surgical findings in patients with obstructive symptoms [4]. The significant advancements in CT technology, along with the progressive use of MDCT in the diagnosis of abdominal emergencies, have determined an increment in the detection of intestinal intussusceptions [6, 7]. The typical bowel-within-bowel appearance [1, 8–10] is often found in asymptomatic patients, with transient intussusception and no underlying disease [11–13]. Although surgical intervention is considered necessary in symptomatic patients with leading point intussusception [14], not every patient with CT evidence of intestinal intussusception may require surgery [15, 16]. The distinction between lead point and non–lead point intussusception, as well as the detection of obstructive complications on MDCT, is important in determining the appropriate treatment, avoiding unnecessary surgery. In this paper, we review the current role of MDCT in the diagnosis and management of intussusception, focusing on features that may guide an accurate selection of adult patients for surgery, both in small bowel and large bowel intussusceptions.

## 2. Anatomy, Pathophysiology, and Classification

Intussusception results from altered intestinal motility, determining the telescoping of one bowel segment (*intussusceptum*) into the lumen of the contiguous intestinal tract (*intussuscipiens*) [1, 8]. Although this invagination can occur anywhere along the gastrointestinal tract, most intussusceptions occur in the junctions between mobile and retroperitoneal fixed intestinal segments.

Intussusceptions can be classified into three types based on the location:

- Enteroenteric, when confined to the small bowel.
- Colocolonic, when involving the large bowel.
- Enterocolonic, which can be ileocaecal or ileocaecocolonic.

According to the literature, ileocaecal intussusceptions are the most common of all the gastrointestinal intussusceptions, followed by enteroenteric intussusceptions, which can account for up to 40% of cases. Colocolonic intussusceptions are the less common type of intussusceptions [11].

Intussusception in an adult can be further classified on the basis of whether a lead point is present. Intussusceptions without a lead point tend to be transient, self-limiting, and nonobstructing. Patients present with nonspecific symptoms if any, like vague abdominal pain. In asymptomatic patients, the diagnosis of intussusception is often an incidental finding on MDCT performed for other reasons [17]. In these cases, most of the time, the small bowel intussusception is self-limited; the length of intussusception is the most reliable predictive indicator of the outcome. Intussusception shorter than 3.5 cm rarely requires surgery [16].

Clinical diagnosis can be difficult even in symptomatic patients with a leading point intussusception, because of the variety of clinical findings at presentation (crampy abdominal pain, nausea, vomiting, and bloody mucoid stools), depending on the underlying cause. Complicated intussusceptions, with bowel wall engorgement due to impaired mesenteric circulation and signs of parietal ischaemia, are associated with a higher risk of perforation and peritonitis [3, 5].

## 3. Role of Imaging

Abdominal MDCT has been shown to be the imaging modality of choice for the detection and assessment of adult bowel intussusception, with a reported accuracy of 58–100% [1, 18]. MDCT is well suited to delineate the presence of the disease and provides valuable information about several features, such as the site of intussusception, the intestinal segments involved, and the extent of the intussuscepted bowel [19]. MDCT has the ability to differentiate between presence and lack of a leading point.

Moreover, MDCT can demonstrate the complications of intussusceptions, represented by bowel wall ischemia and perforation, which are mandatory to promptly refer for surgery.

Merine et al. [15] in 1987 described three CT patterns of intussusception as corresponding to different stages of the disease: the target-like pattern, the reniform pattern, and the sausage-shaped pattern. The first appearance, the target-like pattern, described as a round mass with intraluminal soft-tissue and eccentric fat density, was thought to correspond to an early intussusception with no or minimal obstruction and without signs of ischemia [9]. The second appearance, the reniform pattern, appearing as a bilobed mass with central low attenuation and peripheral higher density, was thought to result from ischemic thickening of the intussusceptum's bowel wall. The latter appearance, the sausage-shaped pattern, was thought to result from alternating areas of low and high attenuation related to the bowel wall, mesenteric fat and fluid, intraluminal fluid, contrast material, or air.

**Figure 1:**
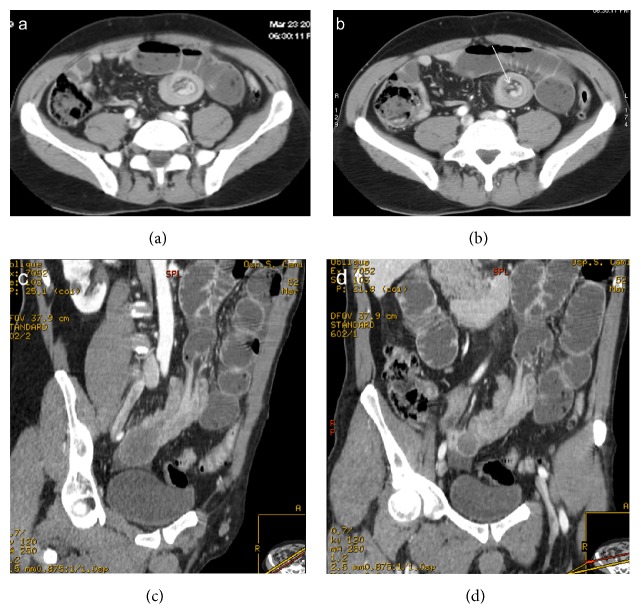
Enteroenteric intussusception. Axial CT images (a, b) demonstrate a round mass with “target” pattern and central hypodense area of mesenteric fat in which vessels are seen as linear enhanced structures (arrow). Oblique CT reformatted images (c, d) oriented parallel to the longitudinal axis of the intussusception depict intussusception as a large “sausage-shaped” mass, showing length of involvement. Gas-fluid levels in the dilated proximal loops are signs of small bowel obstruction.

**Figure 2:**
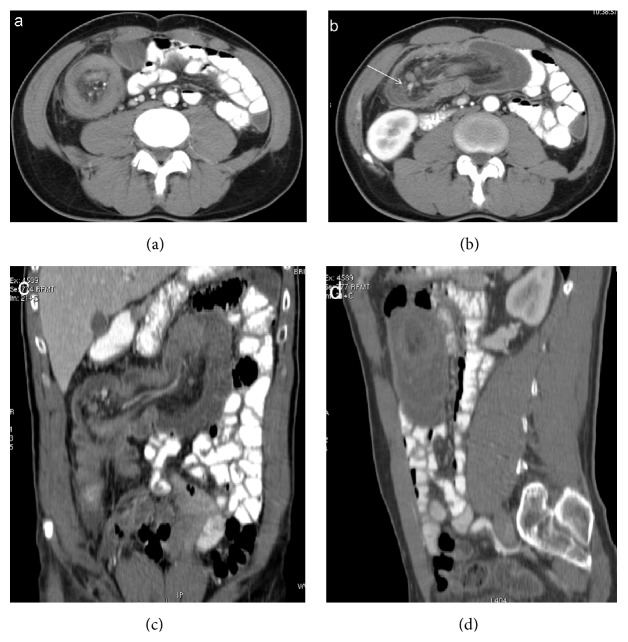
Enterocolonic (ileocaecocolonic) intussusception. Intussusception may present as a target (a), reniform bilobed (b, c), and a sausage-shaped (d) mass depending on the different axial CT scans or reformatted planes. Mesenteric vessels appear as enhanced linear structures between hypodense mesenteric fats (arrow). Dilated proximal bowel loops are opacified with oral contrast.

**Figure 3:**
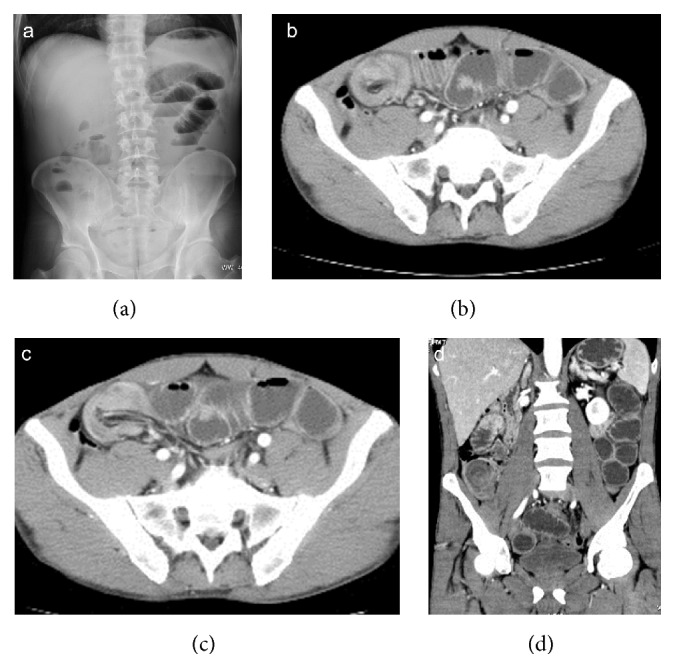
Enterocolonic (ileocaecocolonic) intussusception caused by intestinal lymphoma, with proximal bowel obstruction. Plain abdominal radiography (a) shows gas-fluid levels within distended small bowel loops. Intussusception is well depicted on axial CT scans (b, c) and coronal reformatting (d).

**Figure 4:**
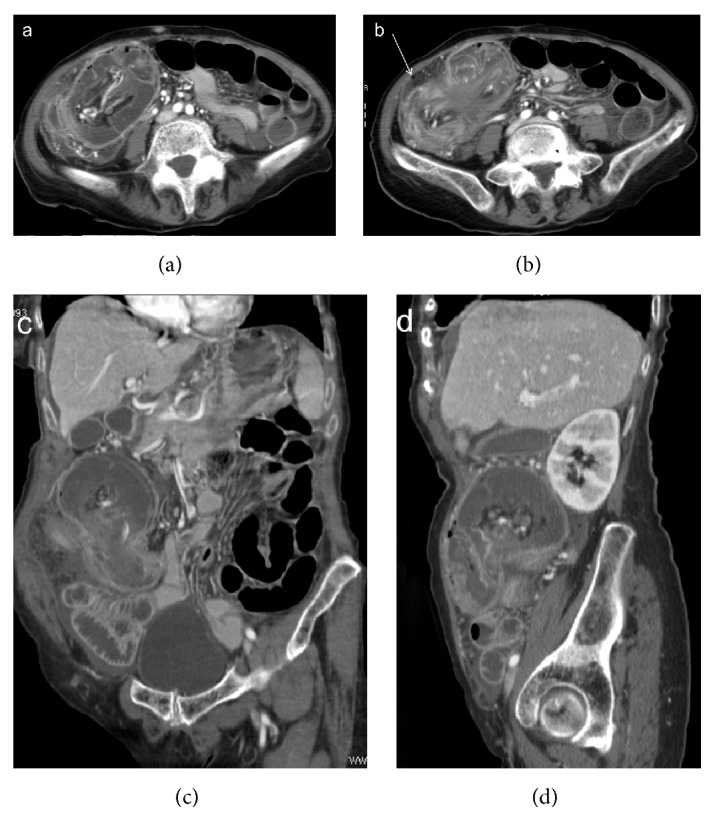
Enterocolic (ileocaecocolonic) intussusception due to a caecal carcinoma. CT images on axial scans (a, b) and oblique reformatting (c, d) show lymph nodes and vascular engorgement in the intussuscepted mesentery and fluid distention of the intussuscipiens. Extraparietal air indicates local perforation (arrow).

Actually, intussusception often appears as a complex soft-tissue mass on MDCT images. It is composed of a central intussusceptum and outer intussuscipiens, separated by mesenteric fat, which appears as a low-attenuation layer. Enhanced vessels are often seen within the mesenteric fat (Figure 1). The image pattern varies according to location, axis of section, bowel wall thickness, and lumen patency. The appearance of an intussusception on CT images is similar to that of a “target” mass when the CT beam is perpendicular to the longitudinal axis of the intussusception and to that of a “sausage” mass when the CT beam is parallel or oblique to the longitudinal axis [10] (Figure 2). The presence of a lead point, the configuration of the lead mass, the degree of bowel wall edema, and the amount of invaginated mesenteric fat all affect the radiological aspect of an intussusception. Intussusception with a lead point usually appears as an abnormal target-like mass with a cross-sectional diameter greater than that of the normal bowel and may be associated with proximal bowel obstruction (Figure 3). It is often not easy to distinguish the distinct anatomic features of the lead mass, because of poor recognisability of the edematous intestinal wall and the lead mass. If there is bowel wall edema due to impaired circulation of the mesenteric vessels, it is difficult to differentiate a lead mass from inflammation because the former may appear amorphous (Figure 4). Even when a leading mass is seen, it is not always possible to reliably distinguish a malignant from benign neoplasm.

## 4. Imaging Features: Enteroenteric Intussusception

Adult enteroenteric intussusceptions are thought to be relatively rare. They can be classified as duodenojejunal, jejunojejunal, or jejunoileal. Duodenojejunal intussusception is rare, because of anatomic fixation of a large portion of the duodenum. Retrograde jejunal intussusceptions, in which retrograde peristalsis determines the telescoping of a distal bowel segment into the adjacent proximal segment, are reported as postoperative complications of Roux-en-Y anastomoses [20].

**Figure 5:**
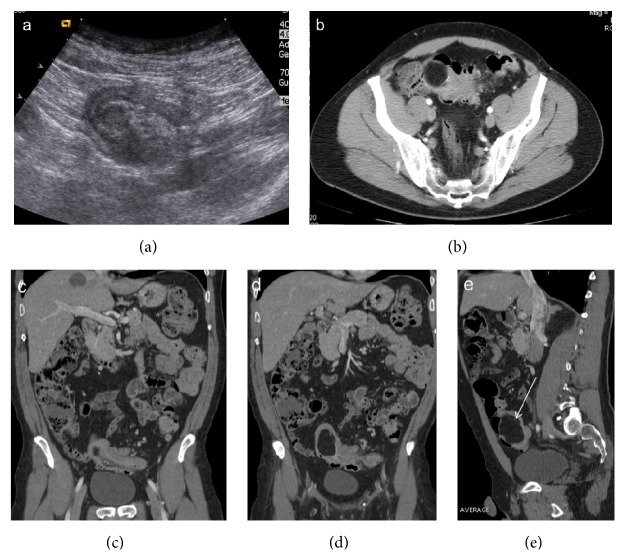
Colocolonic (sigmoid) intussusception caused by a lipoma. Ultrasound scan (a) shows a pelvic layered ovoid mass. CT images on axial (b) and oblique reformatting (c, d) demonstrate an intraluminal lesion with fat attenuation (arrow) that serves as the intussusception lead point.

**Figure 6:**
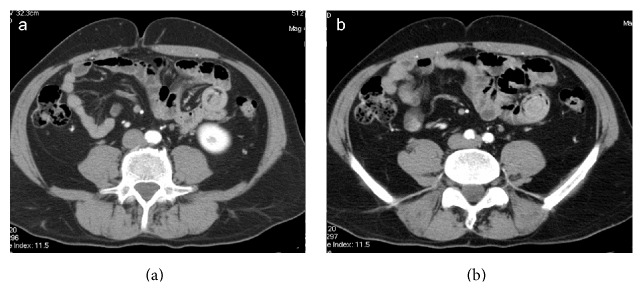
Enteroenteric (ileoileal) transient intussusception in a traumatized patient. Axial CT images oriented perpendicular to the longitudinal plane of the intussusception demonstrate the typical multilayered appearance of small bowel intussusception. Heterogeneous “target” mass with the intussuscipiens, intussusceptum, and vessels within the invaginated mesenteric fat. No signs of significative obstruction, only mild stasis in the small bowel.

**Figure 7:**
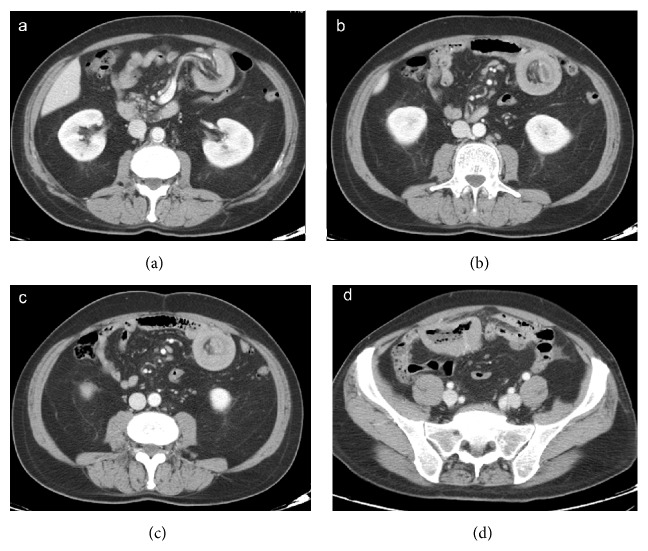
Enteroenteric (ileoileal) intussusception caused by lymphoma. Axial CT images show the typical appearance of small bowel intussusception (a, b, and c). Marked circumferential thickening of the wall of a distal ileum loop (d) is due to lymphoma, which is responsible for intussusception.

Most cases of small bowel intussusceptions are secondary to benign intra- or extraluminal lesions, such as inflammatory lesions, Meckel's diverticulum [21], postoperative adhesions, lipoma (Figure 5), and adenomatous polyps [3], but they can also be iatrogenic (placement of intestinal tube, gastrojejunostomy) or caused by abdominal trauma [22] (Figure 6). Malignant pathologies, accounting for 15% of cases, include adenocarcinoma, malignant GIST, metastasis from various primary sites (lung or breast, malignant melanoma, osteosarcoma, and lymphoma), and primary lymphoma [3] (Figure 7).

**Figure 8:**
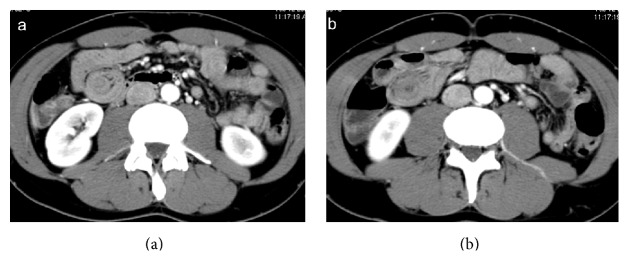
Enteroenteric (ileoileal) intussusception. Target-like mass on axial CT images. Transient intussusception with no signs of intestinal obstruction or intestinal ischemia.

**Figure 9:**
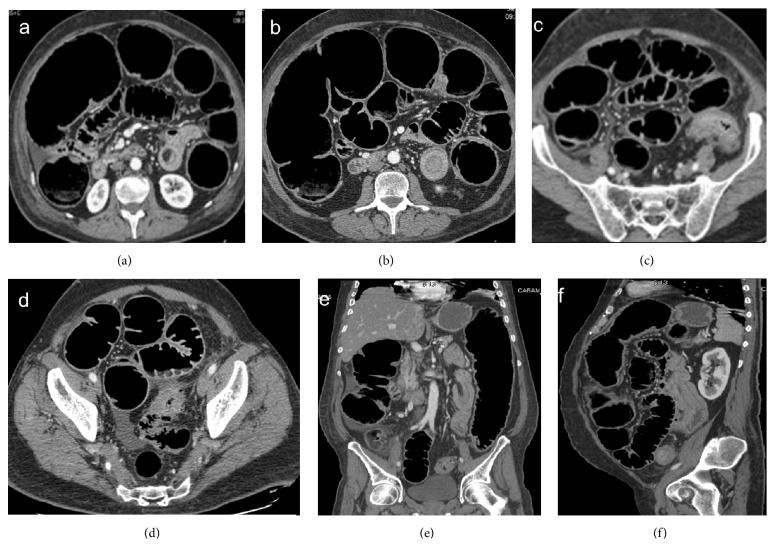
Enteroenteric (ileoileal) intussusception secondary to colic obstruction caused by a sigmoid cancer. Intussusception appears as a small target mass (a, b) in a condition of intestinal obstruction with massive small and large bowel dilatation, due to stenosing sigmoid cancer (d). Coronal (e) and sagittal (f) reformatting better depict the site and the extent of intussusception.

**Figure 10:**
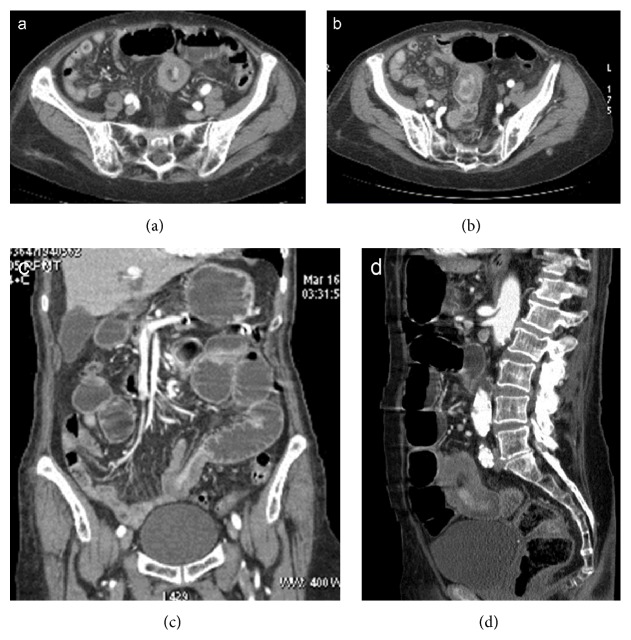
Enteroenteric (ileoileal) intussusception. Bowel wall of intussusception is thickened and enhanced. Signs of small bowel obstruction are seen on axial scans (a, b) and coronal (c) and sagittal (d) reformatting.

Enteroenteric intussusceptions without a lead point tend to be nonobstructing and are usually smaller in transverse diameter and shorter in length than intussusception with a lead point (Figure 8). In some cases, the enteroenteric intussusception is due to increased peristalsis of the intestinal loops caused by distal obstruction, such as a stenosis caused by a neoplasm of the colon (Figure 9). Obstructing enteroenteric intussusceptions, often caused by a lead point, may present at CT with thickening and alterated enhancement of the bowel wall and engorgement of mesenteric vessels (Figure 10).

## 5. Imaging Features: Colocolonic Intussusception

**Figure 11:**
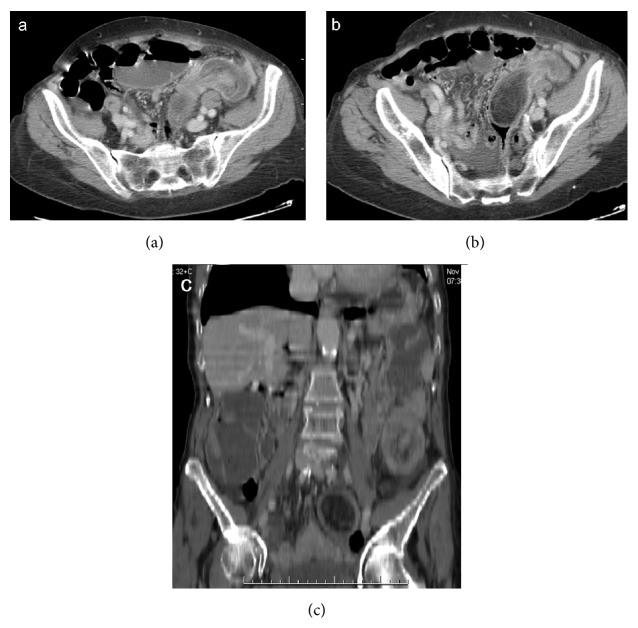
Colocolonic intussusception caused by a sigmoid lipoma. The intussusception is well depicted on axial CT scan (a). Both axial scan (b) and coronal reformatting (c) show the hypodense polypoid mass that acts as the lead point.

Unlike small-bowel intussusception, more than half of large bowel intussusceptions are associated with malignant lesions. Adenocarcinoma is the most common malignant neoplasm associated with colocolonic intussusception, followed by lymphoma and metastatic disease [1]. Among about 30% of large bowel intussusceptions caused by benign lesions, lipomas are the most common cause, followed by GISTs, adenomatous polyps (Figure 11), and other benign conditions like endometriosis and a previous anastomosis [8]. Idiopathic intussusception occurs less often than those of the small bowel, accounting for approximately 10% of intussusceptions [5].

**Figure 12:**
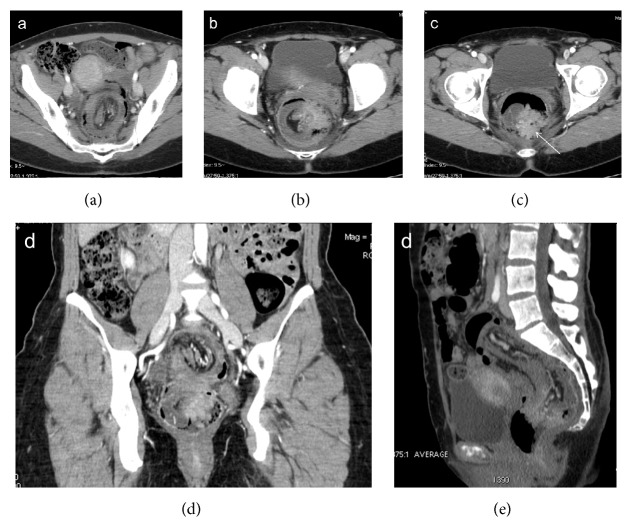
Colocolonic (sigmoid-rectal) intussusception (a) caused by sigmoid adenocarcinoma. The enhanced neoplastic mass, which acts as the lead point, is located in the rectum (arrow), at the tip of intussusceptum (b, c, d, and e).

Sigmoid-rectal intussusception (Figure 12) is a very rare condition in which concentric invagination of distal sigma progresses towards rectal ampulla but does not protrude through the anus [23].

## 6. Imaging Features: Enterocolonic Intussusception

**Figure 13:**
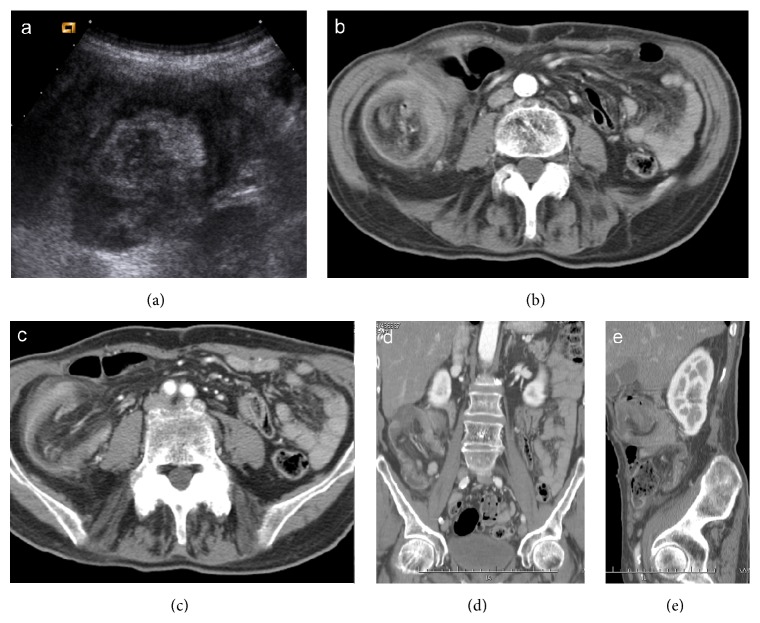
Enterocolic (ileocaecocolonic) intussusception caused by a caecal carcinoma. Ultrasound scan (a) shows a heterogeneous target-like mass located in right flank. CT images on axial scans (b, c) and coronal and oblique reformatting (d, e) demonstrate lead point intussusception with invaginated mesenteric fat, vessels, and lymph nodes.

**Figure 14:**
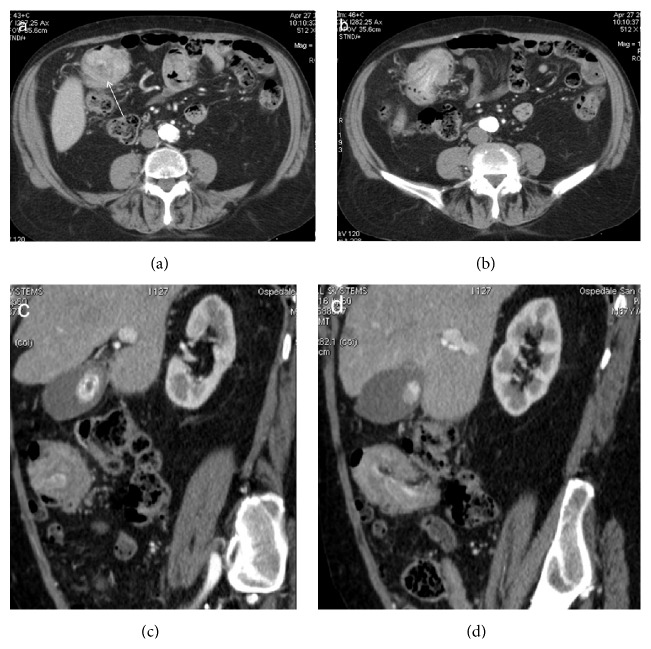
Enterocolonic (ileocaecocolonic) intussusception caused by a polyp. Axial CT images (a, b) demonstrate a soft-tissue density round mass with thin eccentric hypodensity (arrow). Oblique CT reformatting (c, d) clearly shows the enhancement of the lead mass, which facilitates its identification.

In enterocolonic intussusception, the lead point can be located in the small bowel, in the large bowel, often in the caecum (Figure 13), or in the appendix. Enterocolonic intussusception can be further classified as ileocaecal, in which the ileocaecal valve is in site, or ileocaecocolonic (Figure 14), in which the ileocaecal valve is displaced. Appendiceal intussusception is rare and difficult to diagnose radiologically [24].

## 7. Conclusions

Because of significant advancements in MDCT scanners along with increasing use of MDCT in abdominal emergency imaging, the detection of enteroenteric intussusceptions by CT has increased. Intussusceptions are now being detected incidentally on MDCT in patients being scanned for unrelated reasons [8] or in asymptomatic patients, often with transient intussusceptions and without an identifiable lead point. The radiologist can readily make a correct diagnosis, detecting specific MDCT findings such as the bowel-within-bowel appearance. Some findings on CT may be helpful in guiding management and reducing the prevalence of unnecessary surgery. The radiologist's aim is not only to recognize intussusception, but also to define its location, enteroenteric, colocolonic, or enterocolonic, to evaluate underlying pathology, and to identify complicated intussusceptions, associated with obstruction or ischemia, which represent indications for surgical exploration.
